# The Organ Size and Morphological Change During the Domestication Process of Soybean

**DOI:** 10.3389/fpls.2022.913238

**Published:** 2022-06-09

**Authors:** Xuan Zhou, Dongfa Wang, Yawen Mao, Yueqiong Zhou, Limei Zhao, Chunbao Zhang, Yu Liu, Jianghua Chen

**Affiliations:** ^1^CAS Key Laboratory of Tropical Plant Resources and Sustainable Use, CAS Center for Excellence in Molecular Plant Sciences, Xishuangbanna Tropical Botanical Garden, Chinese Academy of Sciences, Kunming, China; ^2^University of Chinese Academy of Sciences, Beijing, China; ^3^School of Life Sciences, University of Science and Technology of China, Hefei, China; ^4^Soybean Research Institute, Jilin Academy of Agricultural Sciences, Changchun, China; ^5^Yunnan Key Laboratory of Plant Reproductive Adaptation and Evolutionary Ecology and Institute of Biodiversity, School of Ecology and Environmental Science, Yunnan University, Kunming, China

**Keywords:** soybean, stem growth habit, leaf size and shape, seed size and weight, domestication

## Abstract

Soybean is one of the most important legume crops that can provide the rich source of protein and oil for human beings and livestock. In the twenty-one century, the total production of soybean is seriously behind the needs of a growing world population. Cultivated soybean [*Glycine max* (L.) Merr.] was domesticated from wild soybean (*G. soja* Sieb. and Zucc.) with the significant morphology and organ size changes in China around 5,000 years ago, including twisted stems to erect stems, small seeds to large seeds. Then it was spread worldwide to become one of the most popular and important crops. The release of the reference soybean genome and omics data provides powerful tools for researchers and breeders to dissect the functional genes and apply the germplasm in their work. Here, we summarized the function genes related to yield traits and organ size in soybean, including stem growth habit, leaf size and shape, seed size and weight. In addition, we also summarized the selection of organ traits during soybean domestication. In the end, we also discussed the application of new technology including the gene editing on the basic research and breeding of soybean, and the challenges and research hotspots in the future.

## Introduction

With the growth of the world food crisis, the plant as the main source of food is particularly important. To increase the crop yield and biomass, breeders used the artificial hybridization approach to select the later generation with significant morphology and organ size changes (Yin et al., 2020). The organ size is also an important parameter for describing organ morphology and characterizing key function genes. However, the molecular mechanism on regulation of plant organ size is also a fundamental question in the field of developmental biology (Yin et al., 2020). Stems, leaves and seeds are three types of important organs which determine the production of plants. All of them are also important agronomic traits during domestication, and cultivars in the same region will be domesticated with similar phenotypes to increase yields or adapt to the environment (Lenser and Theißen, 2013). Increasing the size of plant organs, especially seeds, is one of the most important ways to improve crop yields.

Soybean [*Glycine max* (L.) Merr.] is a major crop for protein and oil production, and is widely grown worldwide. It can provide the rich source of protein and oil for human being and livestock that accounts for approximately 56% of global oilseed production (Zhou et al., 2015; Lu et al., 2016). Recently research showed that global crop production must be doubled in 2050 to meet the growing demands (Foley et al., 2011; Tilman et al., 2011). However, the soybean production is seriously behind the demand of the growing world population, which just has only 1.3% rate of increase per year (Ray et al., 2013). Therefore, increasing yield is an important part of soybean breeding. With the release of soybean reference genome, rapid progresses on functional genes of soybean have been made in the past decade (Zhang et al., 2021). Here, we reviewed functional genes related to yield traits and organ size in soybean, including stem growth habit, leaf size and shape, seed size and weight. Furthermore, we focused on the evolution of three important yield-related traits in soybean during the long-term domestication. In addition, we also discussed the application of new technology including the gene editing on the basic research and breeding of soybean, and the challenges and research hotspots in the future.

## Stem Growth Habit

**FIGURE 1:**
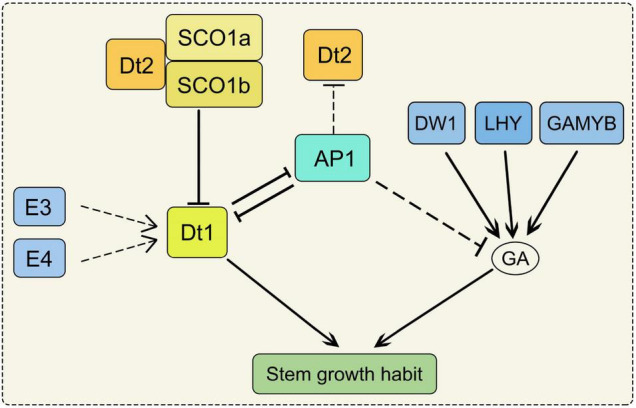
The regulation pathway of stem growth habit in soybean. SOC1a and SOC1b complex interacts with Dt2 to repress the transcription of *Dt1*. AP1 can inhibit *Dt2* expression. Dt1 and AP1 form a suppressive regulatory feedback loop. AP1 may negatively regulate the expression of the GA metabolic pathway-related genes. Phytochromes E3 and E4 induce *Dt1* transcription indirectly. Both of the SOC1, AP1, E3, E4 and Dt2 impact on the stem growth habit through Dt1 in soybean; DW1, LHY, GAMYB could regulate the stem growth habit through GA biosynthetic pathway. SOC1, SUPPRESSOR OF OVEREXPRESSION OF CONSTANS1; AP1, APETALA1; E3 and E4, phytochromes E3 and E4; DW1, a dwarf gene encodes an *ent*-kaurene synthase; LHY, LATE ELONGATED HYPOCOTYL; GA, gibberellin.

Stem growth habit can affect plant height and node number, thus determines grain yield. However, the increase of plant height will incur costs in the construction and maintenance of the stem (Falster and Westoby, 2003). Soybean stem is determined by plant height (PH), node number (NN) and internode length (IL). PH was significantly and positively correlated with NN and IL (Chang et al., 2018; Kou et al., 2021). Both PH and NN affect seed yield by influence on lodging and adaptability of soybean (Assefa et al., 2019). The structure of soybean stems can be divided into three main categories, usually referred to as determinate, semi-determinate and indeterminate (Bernard, 1972). *Dt1* and *Dt2* are two major loci which control the stem growth habit of soybean (Bernard, 1972; Heatherly and Smith, 2004). In the *Dt1Dt1* genetic background, the *Dt2Dt2* genotypes produce semi-determinate phenotypes, but *dt2dt2* genotypes produce indeterminate phenotypes. In the *dt1dt1* genetic background, the phenotype is determinate. These results suggest that the *dt1* allele has an epistatic effect on the expression of the *Dt2*/*dt2* locus (Liu B. et al., 2010; Ping et al., 2014). *Dt1* encodes the GmTFL1b protein which is an ortholog of *Arabidopsis TERMINAL FLOWER1*. PH and IL in the *dt1* mutants were severely reduced under long-day conditions, but there were not obvious variations under short-day conditions (Liu B. et al., 2010). *Dt2* is a gain-of-function of MADS-domain factor which directly binds to the promoter region of *Dt1* to inhibit its transcription (Ping et al., 2014; Liu et al., 2016). *SUPPRESSOR OF OVEREXPRESSION OF CONSTANS1* (*SOC1*) encodes a MIKC^c^-type MADS-box transcription factor which is involved in regulating the flowering time (Lee and Lee, 2010). Loss of function of *GmSOC1* in soybean exhibited delaying the flowering time and increased the PH under both short-day and long-day conditions (Kou et al., 2022). There are two *SOC1* homologs present in soybean (*SOC1a* and *SOC1b*). The GmSOC1a and GmSOC1b complex interacts with Dt2 to repress the transcription of *Dt1* through binding to the *Dt1* promoter, indicating the role of SOC1 in soybean stem growth habit (Figure 1; Liu et al., 2016; Kou et al., 2022). Moreover, Phytochromes E3 and E4 promote PH and NN by inducing *Dt1* transcription indirectly (Figure 1; Xu et al., 2013).

*APETALA1* (*AP1*) encodes a member of MADS-box transcription factors family, and it belongs to the class A gene in the ABC model for floral organ development (Chen et al., 2020). Under short-day conditions, NN and IL were increased in the *gmap1* quadruple mutant, resulting in the increased plant height (Chen et al., 2020). Dt1 and AP1 form a suppressive regulatory feedback loop, and AP1 can inhibit Dt2 expression (Yue et al., 2021). Moreover, AP1 may negatively regulate the expression of the GA metabolic pathway-related genes (Chen et al., 2020). As a member of FLOWERING LOCUS T (FT) family, GmFT5a may repress the expression of *Dt1* by inducing *GmAP1* transcription (Takeshima et al., 2019). Overexpression of *GmFT5a* in wild-type soybean reduced PH, and the same phenotype was obtained by overexpression *GmFT5a* in *DT1*/*dt2* soybean, implying that *GmFT5a* can affect stem growth independently on the *Dt2* (Takeshima et al., 2019). The dwarf gene *GmDW1* encodes an *ent*-kaurene synthase (KS) which regulates the development of plant height in soybean. The *gmdw1* is a gibberellin (GA) biosynthesis-deficient mutant, and the dwarf phenotype is due to the longitudinally decreased cell length (Figure 1; Li et al., 2018). As a MYB transcription factor, *LATE ELONGATED HYPOCOTYL* (*GmLHY*) also impacts PH through the GA biosynthetic pathway. The quadruple mutant of *GmLHY* causes dwarf plants due to defective GA biosynthesis (Figure 1; Cheng et al., 2019; Lu et al., 2020). As a R2R3 MYB transcription factor, overexpression of *GmGAMYB* increases PH in transgenic soybean by up-regulating the expression of *GmGA20ox* (Figure 1; Yang X. et al., 2021). However, another MYB transcription factor showed an opposite function, overexpression of *GmMYB14* reduced PH and IL in soybean (Chen et al., 2021). Moreover, a small ubiquitin-related modifier E3 ligase GmSIZ1 positively regulates the vegetative growth of soybean. *GmSIZ1-RNAi* plants showed a phenotype of reduced leaf size and PH (Cai et al., 2017).

## Leaf Shape and Size

As the energy factory of plants, leaves absorb light energy through photosynthesis and convert it into bioenergy. Because photosynthesis mainly occurs in leaves, the size and shape of leaves have strong effects on the efficiency of photosynthesis, determining the final yield (Gonzalez et al., 2012). Leaves arise at the flank of the shoot apical meristem (SAM), and this process is regulated by multiple gene regulation networks (Efroni et al., 2010). In cultivated soybean, there is a classic phenomenon that narrow leaf cultivars tend to have more seeds per pod than broad leaf cultivars (Jeong et al., 2011). The narrow leaf phenotype is a single-gene recessive inheritance controlled by the *Ln* gene (Dinkins et al., 2002). *Ln* is a homologous gene of *Arabidopsis JAGGED* (*JAG*), which regulates lateral organ development and the number of seeds per pod (NSPP) (Jeong et al., 2013). Interestingly, *ln* widely exists in the high latitude soybean varieties, but it does not exist in the low latitude soybean varieties and is hardly found in wild soybean (Cai et al., 2021). *GmJAG* in low-latitude variety Huachun 6 was edited by CRISPR/Cas9-based genome-editing technology, the offspring showed the same phenotype as that of *ln* mutant which just exists in the high latitude soybean varieties. Furthermore, the yield of *gmjag* was higher than Huachun 6 (Cai et al., 2021). Another gene that can regulate the narrow-leaf phenotype of soybean is *CTP* (*Chicken Toes-Like Leaf and Petalody Flower*). *CTP* is a novel and critical pleiotropic regulator of leaf and flower development. The *ctp* mutant exhibits chicken toes-like leaf and petalody flower. As a novel gene, the function of CTP protein is unclear and valuable to explore in the future. Interestingly, the CTP homologous proteins are only found in the land plant, implying that the *CTP* gene could have played an important role during the evolution of land plants (Zhao et al., 2017). Moreover, overexpression of the flowering repressor gene *E1* could result in small, curly unifoliolate leaves by directly repressing *CINCINNATA (CIN)-like TEOSINTE BRANCHED1/CYCLOIDEA/PROLIFERATING CELL FACTOR* (*TCP*) transcription factor genes (*TCP14*, *TCP29*) (Li Y. et al., 2021). GmKIX8-1 belongs to a family of KIX domain-containing protein, which regulates organ size in soybean. Loss of function of *GmKIX8-1* in soybean leads to big leaf and seed by increased cell proliferation (Nguyen et al., 2021). *YABBY* genes play important roles in lateral organs such as leaves and floral organs. Overexpression of *GmFILa*, a soybean *YABBY* gene, causes curly and long-narrow leaves in *Arabidopsis* (Yang et al., 2019).

## Seed Size and Weight

The seed is one of the most important organs in the plant because the size and number of seeds directly determine the final yield of crops. Seed size is critical to many aspects of evolution and influenced by several key transcription factors (Linkies et al., 2010). *BIG SEEDS1* (*BS1*) encodes a member of group II of the TIFY family of transcription regulators, and it can affect both seed and leaf sizes in legume species (Ge et al., 2016). Down-regulation of soybean *BS1* gene significantly increased the size and weight of seeds and leaves (Ge et al., 2016). *SLB1/MIO1* encodes an Arabidopsis thaliana F-box protein SAP homologous in *Medicago truncatula* (Zhou et al., 2021). SLB1 interacts with BS1 to control organ size, and overexpression of *SLB1* leads to the increased seed and leaf size in soybean (Yin et al., 2020). During the organogenesis of plant, both cell proliferation and cell growth together determine the size of the organ (Liu Z. et al., 2020). GmCIF1 is a cell wall invertase inhibitor in soybean, and *GmCIF1*-RNAi plant significantly improves seed weight and slightly increases seed size (Tang et al., 2017). *GmFAD3*, encoding an omega-3 fatty acid desaturase, can control the leaf and seed size in soybean. *GmFAD3*-silenced plant showed increased seed size and crinkled leaf, and it has been proved to increase seed yield without affecting seed protein or oil content in the greenhouse (Singh et al., 2011). Interestingly, overexpression of *lesquerella* (*Physaria fendleri*) *FAD3* gene in soybean revealed that plant height, total seeds, and total seed weight were significantly increased (Yeom et al., 2020). These seem to imply that *FAD3* is a key gene controlling seed size in soybean and its expression level needs to maintain a balance to control seed size. A *phosphatase 2C-1* (*PP2C-1*) gene contributes to the increase in seed weight/size, and *PP2C-1* may transduce the brassinosteroid (BR) signaling by promoting dephosphorylation of *GmBZRs* which is one of the key transcription factors in BR signaling. Moreover, *GmBZR1* can promote seed weight/size in transgenic *Arabidopsis* for overexpression (Lu et al., 2017). Previous studies have revealed that SWEET proteins play important roles in sugar translocation to seeds, which in turn affects fruit set, and seed composition (Sosso et al., 2015). A knock-out *GmSWEET10* (both *GmSWEET10a* and homolog *GmSWEET10b*) by CRISPR/Cas9 system, exhibited significantly decreased seed size. Inversely, in soybean lines with the overexpression of *GmSWEET10*, seeds are larger than Williams 82 control (Wang et al., 2020).

The cytochrome P450/CYP78A family plays an important role in regulating seed size (Adamski et al., 2009; Wang et al., 2015). Overexpression of *GmCYP78A72* and *GmCYP78A5* in soybean resulted in increased seed size and seed weight (Zhao et al., 2016; Du et al., 2017). Moreover, knock-out of *GmCYP78A72* by CRISPR/Cas9 system does not reduce seed size, but simultaneous silencing of the *GmCYP78A57*, *GmCYP78A70* and *GmCYP78A72* genes reduced seed size. These results suggest that three *GmCYP78A* genes have redundant functions (Zhao et al., 2016). *GmPDAT* plays an important role in increasing seed size. Overexpression of *GmPDAT* results in increased seed size, while RNAi lines have the opposite effect (Liu J. Y. et al., 2020). *GmNAP1* encodes a NCK-associated protein, and loss of function of *GmNAP1* caused the reduced seed size and plant height in soybean (Campbell et al., 2016; Tang et al., 2020). A genome scan for loci involved in soybean domestication identified a semi-dominant locus, *Seed Thickness 1* (*ST1*). *ST1*, encoding a UDP-D-glucuronate 4-epimerase, affects seed size through the pectin biosynthesis pathway. Loss of function of *ST1* by CRISPR/Cas9-based genome-editing technology significantly decreased seed length, width and thickness in soybean (Li et al., 2022).

## Organ Trait Selection During the Domestication Process of Soybean

Domesticated crops play important roles in human nutrition and agriculture. During the domestication of plants, wild populations respond to changing selection pressures and thus adapt to new cultivated ecological niches (Stitzer and Ross-Ibarra, 2018). Recently, some studies showed that cultivated soybeans [*G. max* (L.) Merr.] was domesticated from wild soybean (*G. soja* Sieb. and Zucc.) in China around 5,000 years ago, then it had been introduced to Korea and Japan. Cultivated soybean was introduced to the American continent between the 18th and 20th centuries (Zhou et al., 2015). Nowadays, soybean is widely grown around the world. Phylogenetic studies of whole-genome resequencing data demonstrate the monophyletic nature of domesticated soybean (Lam et al., 2010; Li et al., 2010; Zhou et al., 2015). However, there is a controversy about the domestication center of soybean, and it is mainly assumed that the domestication centers are the Yellow River of China (Li et al., 2010; Han et al., 2016; Wang et al., 2016). Furthermore, soybean populations are geographically structured, and some agronomic traits show geographic distribution patterns (Zhou et al., 2015). For example, soybean varieties from northern China tend to exhibit narrow leaf shapes, while varieties from southern China exhibit ovate leaf shapes (Zhou et al., 2015; Cai et al., 2021). The key regulatory gene responsible for this phenomenon may be *Ln*, which was not found in 383 re-sequenced accessions collected from low latitudes (Cai et al., 2021). Moreover, *Dt1*, a stem determinacy regulatory factor, showed a strong regional differentiation, as the frequency of mutant alleles gradually increased from northern to southern regions (Zhou et al., 2015).

During the soybean domestication process, the plant morphology has undergone tremendous changes. Wild soybean has much smaller seeds than cultivated soybean, and it also has procumbent and twining stems, breakable pods and impermeable seed coats (Chen and Nelson, 2004). These significant changes contribute to the growth adaptation and yield improvement of soybean. Therefore, some traits and their potential genes were selected and inherited during geographical differentiation or local breeding. Seed size as an important agronomic trait has been selected during crop domestication (Linkies et al., 2010; Ge et al., 2016). Compared to wild soybean, cultivated soybean seeds are longer, wider and thicker (Li et al., 2022). During the transition of soybean seeds from flat to round shape, *ST1* as a key factor was strongly selected to increase seed size (Li et al., 2022). The *SoyWRKY15a* gene is differentially expressed in wild soybean and cultivated soybean, and this gene may be involved in the regulation of seed size and possibly in the domestication process of soybean (Gu et al., 2017). Recently, based on whole-genome resequencing data, *GmSWEET10a* was found to undergo a selection process during the soybean domestication (Wang et al., 2020). *GmCYP78A10* has undergone significant selection during domestication. The *GmCYP78A10a* allele has been eliminated in cultivated soybean at the early stage of soybean breeding, while the *GmCYP78A10b* allele accumulated and became the dominant allele in cultivated soybean (Wang et al., 2015). However, some useful genetic loci may be lost during domestication in cultivated soybean. During the process of domestication, 70% of wild soybean genes were lost in cultivated soybean (Zhuang et al., 2022). Therefore, we can try to “pick up” the key function genes, which control good traits in wild soybean and lose in cultivated soybean, and apply them on molecular breeding to achieve the purpose of improving yield. Whole-genome sequencing analysis of wild and cultivated soybean uncovered 183 genomic rearrangements that affect important phenotypic traits such as flowering time, disease resistance, and stress tolerance. The genetic diversity of wild soybean could provide a resource for soybean breeding (Zhuang et al., 2022).

## Concluding Remarks and Future Perspectives

**FIGURE 2:**
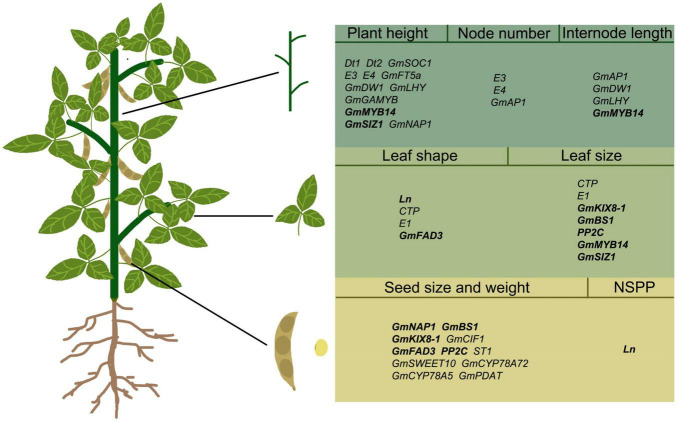
Regulatory genes in different organs of soybean. The words in bold refer to genes that simultaneously regulate the morphology of two soybean organs. Simultaneous regulation of stem and leaf: *GmMYB14* and *GmSIZ1*; Simultaneous regulation of leaf and seed: *Ln*, *GmKIX8-1*, *GmBS1*, *GmFAD3*, *PP2C*; NSPP, number of seeds per pod.

Over the past 20 years, understanding of soybean organ development has grown, and the regulatory genes for many key traits have been identified in soybean (Table 1 and Figure 2), such as *Dt1* and *Dt2* for stem growth habit (Liu B. et al., 2010; Ping et al., 2014), *Ln* for narrow leaf (Jeong et al., 2013), and *GmSWEET10* for seed size (Wang et al., 2020), etc. However, there are still many QTLs controlling organ development in soybean that have not been isolated and confirmed. In other species, the regulation mechanism of organ size has been studied in depth, but the research of functional genes in soybean has been fallen behind. The power of gene knock-out and RNAi-silence technologies has given a new way to study these functional genes in soybean. Targeted silencing of some possible key trait genes can explore their unknown functions in soybean. However, there are many redundant genes in soybean genome, so it is very difficult to study gene function by CRISPR/cas9 technology. Furthermore, Pan-genome sequencing and re-sequencing technologies can link genetic variation to candidate genes that determine important traits (Liu Y. et al., 2020).

**TABLE 1:** Published function genes regulating the organ size and shape of soybean.

Gene name	Gene ID	Type	Stem phenotype	Leaf phenotype	Seed phenotype	References
*Dt1/GmTFL1b*	Glyma.19g194300	LOF	Decreased PH under long-day conditions; No difference PH under short-day conditions			Liu B. et al., 2010;
*Dt2*	Glyma.18g273600	GOF	indeterminate phenotypes			Bernard, 1972; Ping et al., 2014
*GmSOC1*	Glyma.18G224500 (a) Glyma.09G266200 (b)	LOF	Increased PH			Kou et al., 2022
*E3/*G*mPhyA3*	Glyma.19g224200	LOF	Increased PH and NN			Xu et al., 2013
*E4*	Glyma.20g090000	LOF	Increased PH and NN			Xu et al., 2013
*GmAP1*	Glyma.16g091300 (a) Glyma.08g269800 (b) Glyma.01g064200 (c) Glyma.02g121600 (d)	LOF	Increased PH, NN and IL under short-day conditions			Chen et al., 2020; Kou et al., 2021
		OE	Decreased PH			Chen et al., 2020
*GmFT2a*	Glyma.16g150700	CRISPR/Cas9-mediated	Increased PH			Li X. et al., 2021
		OE	Decreased PH under short-day conditions			Takeshima et al., 2019
*GmFT5a*	Glyma.16g044100	CRISPR/Cas9-mediated	Increased PH			Li X. et al., 2021
		OE	Decreased PH			Takeshima et al., 2019
*GmDW1*	Glyma.08g163900	LOF	Decreased PH and IL			Li et al., 2018
*GmLHY*	Glyma.16g017400 (1a) Glyma.07g048500 (1b) Glyma.19g260900 (2a) Glyma.03g261800 (2b)	CRISPR/Cas9-mediated	Decreased PH and IL			Cheng et al., 2019
*GmGAMYB*	Glyma.13g187500	OE	Increased PH			Yang X. et al., 2021
*GmMYB14*	Glyma.19g164600	OE	Decreased PH and IL	Decreased		Chen et al., 2021
*GmSIZ1*	Glyma.12g071300 (a) Glyma.11g154005 (b)	RNAi	Decreased PH	Decreased		Cai et al., 2017
*Ln/GmJAG1*	Glyma.20g116200	LOF		Narrow leaf	Increased NSPP	Jeong et al., 2013
*CTP*	Glyma.05g022400	LOF		chicken toes-like leaf	None seeds	Zhao et al., 2017
*E1*	Glyma.06g207800	OE		small, curly unifoliolate leaves		Li Y. et al., 2021
*GmKIX8-1*	Glyma.17g112800	LOF		Increased	Increased	Nguyen et al., 2021
*GmBS1*	Glyma.10g244400	RNAi		Increased	Increased	Ge et al., 2016
*GmCIF1*	Glyma.17g036300	RNAi			significantly Increased weight and slightly increased seed size	Tang et al., 2017
*GmFAD3*	Glyma.03g056700 (a) Glyma.07g151300 (b) Glyma.11g174100 (c)	RNAi		Crinkled leaf	Increased	Singh et al., 2011
*PP2C*	Glyma.17g221100	OE		Increased	Increased	Lu et al., 2017
*GmSWEET10*	Glyma.15g049200 (a) Glyma.08g183500 (b)	CRISPR/Cas9-mediated			Decreased	Wang et al., 2020
		OE			Increased	Wang et al., 2020
*GmCYP78A72*	Glyma.19g240800	OE			Increased	Zhao et al., 2016
*GmCYP78A5*	Glyma.05g019200	OE			Increased	Du et al., 2017
*GmPDAT*	Glyma.13g108100	RNAi			Decreased	Liu J. Y. et al., 2020
		OE			Increased	Liu J. Y. et al., 2020
*GmNAP1*	Glyma.20g019300	LOF	Decreased PH		Decreased	Campbell et al., 2016; Tang et al., 2020
*ST1*	Glyma.08g109100	CRISPR/Cas9-mediated			Decreased	Li et al., 2022

*LOF, loss of function; GOF, gain of function; OE, overexpression; PH, plant height; NN, node number; IL, internode length; NSPP, number of seeds per pod; Gene ID is from Williams 82.a4.v1.*

In the future, soybean breeding is still an important but challenging work. Seed size is a key trait in breeding high-yielding soybeans, but there is a balance between seed size and yield. However, *GmFAD3*-silenced soybean showed both the larger seed and increased yield phenotype without affecting fat and protein content (Singh et al., 2011). It predicts that *GmFAD3* may be a key factor in improving soybean yield. In addition, stem and leaf shape are also another key factors in improving soybean yield. Moderately increased planting densities have boosted crop yield. The vertical plant structure benefits from dense planting (Tian et al., 2019). The good morphology combined with proper planting density can effectively increase yield (Liu S. et al., 2020). Therefore, stem growth habit has been the focus of soybean domestication, and there are many QTLs for plant height waiting for our further identification (Yang Q. et al., 2021). Furthermore, resequencing data of wild and cultivated soybean revealed many QTLs related to agronomic traits, which provided the powerful database for the research of functional genes in the future (Lam et al., 2010; Zhou et al., 2015). The orderly integration of different superior traits with molecular tools will be one of the important challenges for soybean breeding in the future.

## Author Contributions

XZ, DW, YM, YZ, LZ, CZ, and JC drafted the manuscript. JC conceived to the article and revised the manuscript. All authors contributed to the article and approved the submitted version.

## Conflict of Interest

The authors declare that the research was conducted in the absence of any commercial or financial relationships that could be construed as a potential conflict of interest.

## Publisher’s Note

All claims expressed in this article are solely those of the authors and do not necessarily represent those of their affiliated organizations, or those of the publisher, the editors and the reviewers. Any product that may be evaluated in this article, or claim that may be made by its manufacturer, is not guaranteed or endorsed by the publisher.
